# Intraocular Pressure Measurement Using Ocular Response Analyzer, Dynamic Contour Tonometer, and Scheimpflug Analyzer Corvis ST

**DOI:** 10.1155/2019/3879651

**Published:** 2019-10-16

**Authors:** Lisa Ramm, Robert Herber, Eberhard Spoerl, Frederik Raiskup, Lutz E. Pillunat, Naim Terai

**Affiliations:** Department of Ophthalmology, University Hospital Carl Gustav Carus, TU Dresden Fetscherstraße 74, 01307 Dresden, Germany

## Abstract

**Purpose:**

To compare intraocular pressure (IOP) measurements with Goldmann applanation tonometry (GAT), ocular response analyzer (ORA), dynamic contour tonometer (DCT), and Corvis ST (CST) in healthy subjects.

**Methods:**

In a prospective, observational study, IOP measurements with GAT (GAT-IOPc), ORA (IOPcc), DCT (DCT-IOP), and CST (bIOP) were performed and analyzed in 94 healthy subjects.

**Results:**

Mean age of the participants was 45.6 ± 17.2 years (range 18 to 81 years). Mean GAT-IOPc was 12.9 ± 2.4 mmHg, mean DCT-IOP was 16.1 ± 2.6 mmHg, and mean IOPcc was 15.6 ± 3.3 mmHg. DCT-IOP and IOPcc were significantly higher than GAT-IOPc (*P* < 0.001). Mean bIOP was 13.5 ± 2.4 mmHg that was slightly higher but not significantly different from GAT-IOPc (*P*=0.146). Correlation analysis of IOP values and central corneal thickness (CCT) revealed a negative correlation between GAT-IOPc and CCT (*r* = −0.347; *P*=0.001). However, IOPcc, DCT-IOP, and bIOP showed no significant correlation to CCT. Only bIOP revealed a weak but significant age dependency (*r* = 0.321, *P*=0.002).

**Conclusion:**

All tonometry devices showed a good agreement of biomechanical corrected IOP values with GAT-IOPc. As no influence of CCT on IOPcc, DCT-IOP, and bIOP was detectable, the used correction algorithms appear to be appropriate in these tonometers in the clinical setting. The highest agreement was found between GAT-IOPc and bIOP. However, bIOP weakly correlated with participants' age. Further studies are needed to elucidate the role of bIOP for IOP measurement.

## 1. Introduction

Glaucomatous optic neuropathy is one of the leading causes of irreversible blindness worldwide [1, 2]. Estimates suggest that there are 60 to 80 million people affected [1, 3, 4]. Different studies identified intraocular pressure (IOP) as the leading risk factor for glaucoma development and progression as well as the most important therapeutic target [5–12]. Therefore, it becomes clear that precise IOP estimations are crucial for proper management of these patients.

For many years, Goldmann applanation tonometry (GAT) is the gold standard for IOP measurement [2,13–15]. Despite a broad acceptance, uncertainties result from different influencing factors. Sources of error are, for example, an inappropriate fluorescein pattern, corneal changes like edema or astigmatism, pressure on the globe during the examination, IOP oscillations due to ocular perfusion, incorrect calibration, and various other factors [13, 15]. In addition, corneal properties such as thickness, curvature, rigidity, corneal tear film adhesion, age, and medical history play an important role in the correct IOP estimation [13, 14, 16]. For this reason, different new tonometers, accounting for thickness and other biomechanical properties of the cornea, are available at present.

The Pascal Dynamic Contour Tonometer (DCT, Ziemer Ophthalmic Systems AG, Port, Switzerland), the Reichert Ocular Response Analyzer (ORA, Reichert, Delpew, NY, USA), and the Dynamic Scheimpflug Analyzer Corvis ST (CST, Oculus, Wetzlar, Germany) are three relatively new methods designed to be less affected by corneal biomechanical properties.

Briefly, the DCT is a contact method which is mainly based on the principle that by matching the contour of the cornea, the pressure on the outside correlates to the pressure on the inside of the globe [17]. In contrast, the ORA is a noncontact approach and uses a dynamic bidirectional applanation process of the cornea to measure its biomechanical properties and the IOP [18]. The newest noncontact tonometer is the CST. Also, during CST measurement, a corneal applanation is induced and a Scheimpflug camera device is used to evaluate the dynamic response of the cornea and to measure the IOP [19–21].

Before introducing these newer instruments into the clinical setup, their correlation with GAT should be evaluated. Therefore, in the present study, these tonometers were used in healthy subjects and measurement results of corrected GAT-IOP (GAT-IOPc) were compared to IOP values determined by DCT (DCT-IOP), ORA (IOPcc), and CST (bIOP).

## 2. Methods

This prospective study was conducted at the Department of Ophthalmology of the University Hospital Carl Gustav Carus, Technical University Dresden, Germany. The study protocol was approved by the local ethics committee and followed the Declaration of Helsinki. All subjects signed a written informed consent before participation. Inclusion criteria were age over 18 years and the absence of any corneal pathology, previous ocular surgical interventions or injuries, contact lens wear, glaucoma, and systemic connective tissue diseases. Ninety-six healthy voluntary subjects were recruited. Participants had to pass a complete ophthalmic examination including slit lamp biomicroscopy, GAT, and fundus biomicroscopy. GAT measurements were taken as one reading performed by an experienced investigator according to standard protocols [22, 23]. Consecutive measurements by DCT, ORA, and CST were done. These investigations were taken in a sitting position and in both eyes of the subjects (first the right eye, subsequently the left eye). The fixed sequence of examinations in all patients was (1) ORA, (2) CST, (3) DCT, and (4) GAT. Hereby, GAT was the last measurement to avoid a possible influence of riboflavin on following investigations. According to reports of previous studies, a break of 10 min between the applications of each of the different devices was taken to provide optimal starting conditions for the next measurement and to eliminate measurement bias [24, 25]. In case of repeated investigations with the same device (ORA, DCT), a further 10-minute break between the individual measurements was not considered necessary. The quality of the measurements had to reach a “WS > 5” (waveform score) in the ORA, an “ok” in the CST, and a “Q1” to “Q3” in the DCT. One eye was randomly chosen for further analysis. GAT values were corrected using the formula GAT-IOPc = GAT-IOP + (−0.0423 × CCT) + 23.28 [26]. Central corneal thickness (CCT) was provided by the Pentacam (Oculus, Wetzlar, Germany).

### 2.1. Dynamic Contour Tonometry (DCT, Ziemer Ophthalmic Systems AG, Switzerland)

The DCT is a contact tonometer using a piezo-resistive sensor tip attaching the cornea concavely while the curvature of the cornea is not affected. Therefore, the natural shape of the cornea is maintained during measurement and a smaller influence of corneal biomechanical properties than in GAT is considered. Details of the measurement principle were described elsewhere [17], and a good repeatability and reproducibility of the DCT have been confirmed in the past [27]. The mean value of two consecutive DCT-IOP measurements was used for further analysis.

### 2.2. Ocular Response Analyzer (ORA, Reichert Ophthalmic Instruments, Depew, NY)

The ORA is a noncontact tonometry device. During the measurement, a rapid jet of air of increasing intensity induces a bidirectional applanation process of the cornea. An infrared beam is used to monitor the corneal deformation. From the differences of the acting pressures to achieve defined corneal deformation states, the device calculates and provides four variables: the Goldmann-correlated IOP (IOPg), the corneal-compensated IOP (IOPcc), the corneal resistance factor (CRF), and the corneal hysteresis (CH). Details are described elsewhere [18, 23, 28]. In the present study, for every eye, three consecutive measurements were obtained and the average (calculated by the ORA software) was used for further analysis. Measurements below a waveform score of five were excluded due to insufficient quality.

### 2.3. Dynamic Scheimpflug Analyzer Corvis ST (CST, Oculus, Wetzlar, Germany)

The CST measures the IOP and evaluates the dynamic response of the cornea to an air puff. During measurement, a two-dimensional image of the cross section of the deforming cornea is created using a high-speed Scheimpflug camera and amplitude, duration, and velocity of the corneal applanation are recorded [19–21, 29]. The biomechanical-corrected IOP (bIOP) represents a correction algorithm and obtains IOP estimates that are less affected by the main corneal stiffness parameters and age, removing the dependency on a major error source and producing more reliable IOP estimations for glaucoma management [29, 30]. In the present study, the latest software of CST (1.3r15389) was used to assess the bIOP. Measurements with the CST were only taken once in every eye since previous reports described reliable and good-quality results even after a single measurement time point [31, 32].

### 2.4. Statistical Analysis

All data were compiled using a spreadsheet software (Microsoft Office Excel 2013, Microsoft Corporation, Redmond, WA) and transferred to SPSS Version 24 (IBM Statistics; New York, USA). IOP values of all tonometry devices were normally distributed (Shapiro–Wilk test) and analyzed using ANOVA for repeated measurements. Mean IOP values, standard deviations of the means, and the amount of IOP differences between the tonometry devices were indicated. Correlations between IOP and CCT as well as between IOP and age were assessed using Pearson correlation analysis. A *P* value <0.05 was considered as statistical significant. Bland–Altman plots were designed using MedCalc (software version 17.6; Belgium).

## 3. Results

The present study initially included 96 eyes of 96 healthy subjects. Two eyes were excluded from further analysis due to incomplete data acquisition. Mean age of the remaining 94 participants was 45.6 ± 17.2 years (range 18 to 81 years). Thirty-nine subjects were female, and 55 were male. Mean CCT was 553 ± 33 *μ*m, and mean GAT-IOPc was 12.9 ± 2.4 mmHg. Mean DCT-IOP was 16.1 ± 2.6 mmHg, which was significantly higher than GAT-IOPc (*P* < 0.001). Also, IOPcc was significantly different from GAT-IOPc (*P* < 0.001) and showed a mean value of 15.6 ± 3.3 mmHg. Mean bIOP was 13.5 ± 2.4 mmHg and also higher but not significantly different from GAT-IOPc (*P*=0.146). Data are summarized in Tables 1 and 2.

**Figure 1:**
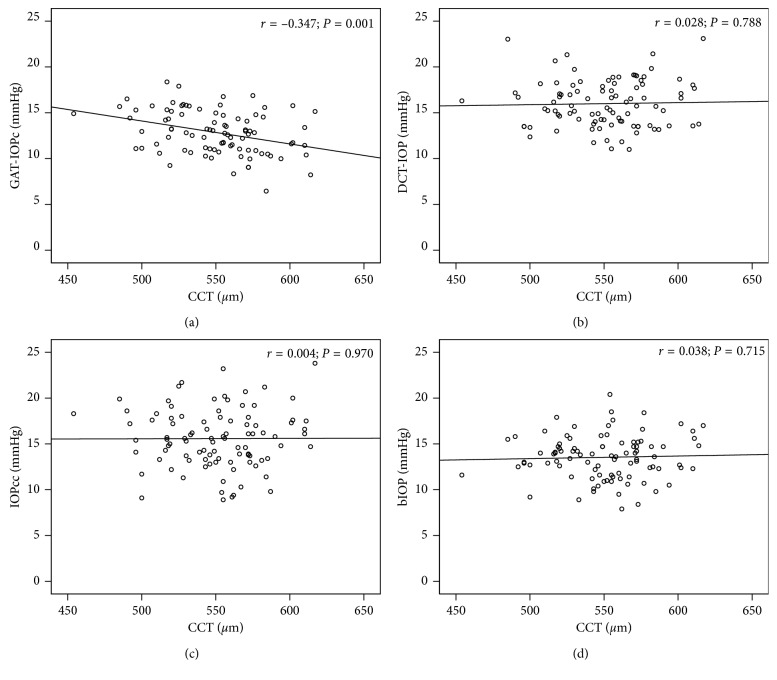
Correlation analysis between central corneal thickness (CCT) and intraocular pressure (IOP) measurements using (a) Goldmann applanation tonometry (GAT-IOPc), (b) dynamic contour tonometry (DCT-IOP), (c) ocular response analyzer (IOPcc) and (d) Corvis ST (bIOP); *r* = Pearson correlation coefficient.

Correlation analysis revealed a significant association between GAT-IOPc and CCT (*r* = −0.347, *P*=0.001, Figure 1(a)). The IOP measurements by ORA (IOPcc), CST (bIOP), and DCT (DCT-IOP) showed no significant correlation to CCT (Figures 1(b)–1(d)).

**Figure 2:**
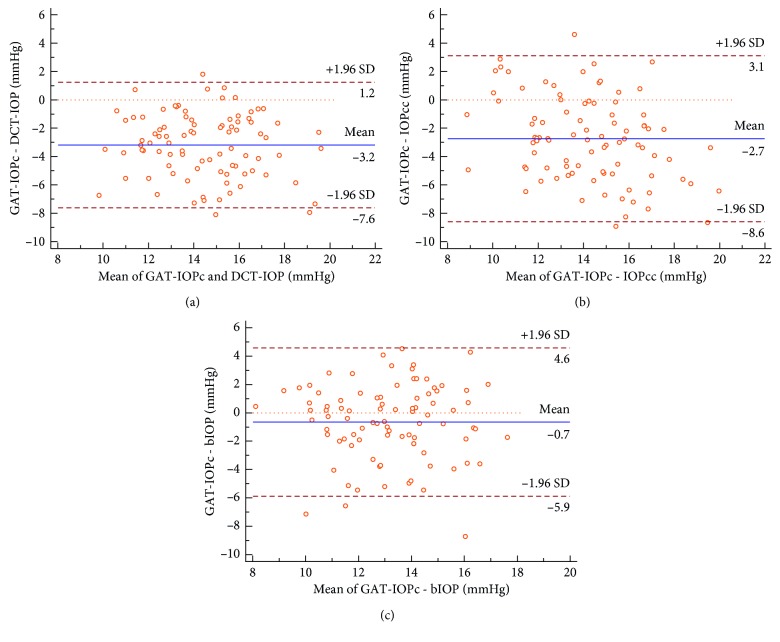
Bland–Altman analyses between mean intraocular pressure (IOP in mmHg) measured by (a) Goldmann applanation tonometry (GAT-IOPc) and dynamic contour tonometry (DCT-IOP), (b) ocular response analyzer (IOPcc) and (c) Corvis ST (bIOP).

Although DCT-IOP was significantly higher than GAT-IOPc, results of both devices showed a good correlation (*r* = 0.594, *P* < 0.001). The 95% limits of agreement ranged from 1.2 mmHg to −7.6 mmHg (Figure 2(a)). IOPcc and GAT-IOPc showed a good correlation despite the significant difference of their means, too. The 95% limits of agreement ranged from 3.1 mmHg to −8.6 mmHg (Figure 2(b)). The mean gap between bIOP and GAT-IOPc was the lowest and showed no significance. However, the correlation was not as pronounced as for the results of the other tonometers (*r* = 0.379, *P* < 0.001). The 95% limits of agreement ranged from 4.6 to −5.9 mmHg (Figure 2(c)).

**Figure 3:**
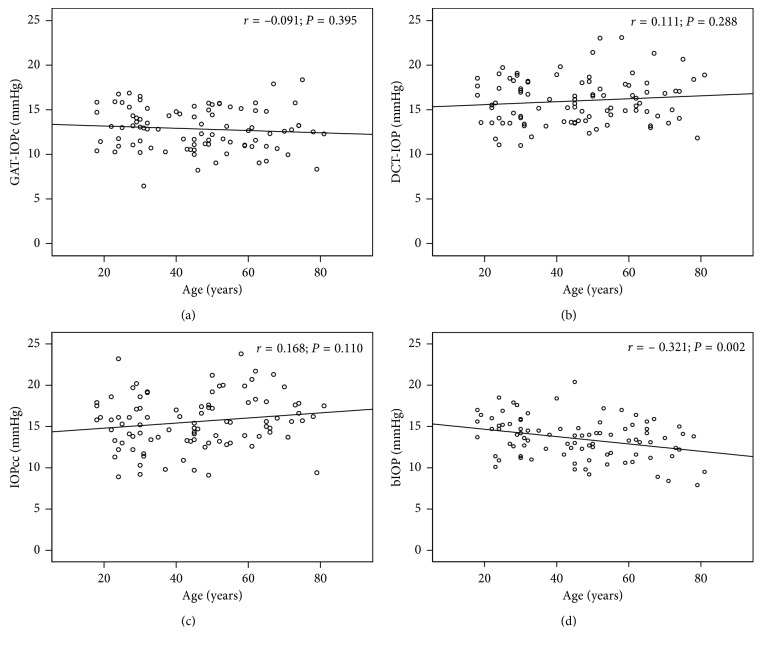
Correlation analysis between age and intraocular pressure (IOP) measurements using (a) Goldmann applanation tonometry (GAT-IOPc), (b) dynamic contour tonometry (DCT-IOP), (c) ocular response analyzer (IOPcc), and (d) Corvis ST (bIOP); *r* = Pearson correlation coefficient.

GAT-IOPc did not correlate to age (*r* = 0.091, *P*=0.395, Figure 3(a)), and DCT-IOP and IOPcc showed no significant age dependency, too (*r* = 0.111, *P*=0.288, Figure 3(b) and *r* = 0.166, *P*=0.110, Figure 3(c)). In contrast, a weak but statistically significant correlation between bIOP and age was found (*r* = −0.321, *P*=0.002, Figure 3(c)).

## 4. Discussion

In the present study, the agreement of GAT-IOPc measurements and IOP values determined by DCT, ORA, and CST was investigated.

As GAT is considered as the gold standard for IOP measurement, we detected a mean GAT-IOPc of 12.9 ± 2.4 mmHg as a reference value in our cohort of healthy subjects. This value lies within the range of a healthy normative population [2, 15]. The standard deviation of the mean, which is regarded as an indicator of a reliable value assessment, conforms to findings of previous studies [17, 33–36]. However, GAT could also be afflicted with errors. As mentioned above, inappropriate fluorescein pattern, pressure on the globe, IOP oscillations, incorrect device calibration, or corneal tear film adhesion may play a role. Furthermore, corneal thickness and biomechanical properties seem to have a strong influence on GAT-IOP [13–16]. Kohlhaas et al. recommended 1 mmHg IOP correction for every 25 *μ*m deviation of CCT from 550 *μ*m [26]. Other authors suggested 2 or 3 mmHg for a 50 *μ*m CCT deviation from 535 *μ*m [37] or 2 mmHg correction for each 100 *μ*m CCT change [38]. In the current study, GAT-IOP was corrected according to the formula published by Kohlhaas et al. [26]. However, as GAT-IOPc still weakly correlated to CCT, an incorrectness cannot be ruled out. The main problem is that true IOP is not known and it can only be investigated by an intracameral measurement, which is not practicable in clinical practice. To remedy this problem, new tonometers accounting for corneal factors have been developed and should be discussed below.

The consistency of DCT-IOP and GAT-IOP was investigated in earlier studies. The reported mean differences of 1.6 ± 2.1 mmHg found by Kouchaki et al., 1.8 mmHg observed by Cook et al., and 1 mmHg reported by Pache et al. were lower than the deviation found in the present study [13, 35, 39]. In agreement with our result, previous investigations showed constant higher DCT-IOP in comparison with GAT-IOP. According to Pache et al., the reason might be that DCT was calibrated with a manometrically controlled pressure standard instead with GAT [39]. Furthermore, Lee et al. reported a CCT-dependency of DCT-IOP in case of CCT over 550 *μ*m [40], which also might contribute to a higher average DCT-IOP.

For ORA measurement, Cook and colleagues reported a smaller mean difference of 1.5 mmHg between the uncorrected ORA-IOP and the GAT-IOP [13]. Goldich and coworkers detected a difference of 2.4 ± 2.6 mmHg between IOPcc and GAT-IOP [41], which is similar to our result, and Kouchaki et al. reported a mean deviation of 0.8 ± 2.3 mmHg [35]. Overall, IOPcc overestimated GAT-IOP in these studies and reasons remain speculative. IOPcc calculation algorithm was initially designed for eyes with reduced CCT to provide reliable IOP measurement before and after refractive surgery [42]. This fact might contribute to deviations between GAT-IOPc and IOPcc. The scattering of IOPcc and DCT-IOP values in the current study complied with the results of earlier investigations [1, 17, 35, 41, 43, 44].

However, we were able to detect a distinct lower deviation of bIOP from GAT-IOPc. Unfortunately, data about bIOP assessment are limited at present. Vinciguerra et al. recently published a comparison of bIOP and GAT-IOP in primary open-angle glaucoma, ocular hypertension (OHT), and healthy subjects [45]. In this study, bIOP was significantly lower than GAT-IOP and CCT-adjusted GAT-IOP in controls and OHT, but bIOP was significantly higher in glaucoma patients [45]. This is contrary to our result of a nonsignificant higher bIOP than GAT-IOPc in healthy subjects. On the other hand, a study published by Eliasy et al. found a good accordance between IOP and bIOP assessed in ex vivo human eyes. The reported IOP difference (0.3 ± 1.6 mmHg) approximates to our result, and the main advantage of the study by Eliasy et al. is the knowledge of the true IOP value in ex vivo setting [46]. According to the calculation formula of bIOP by Joda et al. [30], the scattering in the current study is comparable to the absolute IOP values and consistent with findings of previous studies [1, 17, 29, 36, 44].

Although DCR-IOP [13, 35, 39], IOPcc [7, 47, 48], and previous reported uncorrected CST-IOP values [17, 36, 49] might overestimate GAT-IOPc, an overall good consistency between GAT-IOPc and biomechanically corrected IOP measurements with DCR and ORA was found. Nevertheless, the mean difference between GAT-IOPc and bIOP in a previous study [46] and the current study significantly undercuts the results of the other tonometers. The reasons might be the underlying measurement method in each case as well as the used correction formulas. The CST-IOP is assessed during a measurement process accounting for corneal biomechanical properties (as DCT-IOP and IOPcc), but bIOP further includes a calculated correction for age and CCT, which might increase its correctness. In agreement, Vinciguerra et al. assumed that bIOP may have higher accuracy and repeatability of measurements in comparison with GAT [45].

There is no doubt about the influence of corneal thickness and biomechanics on IOP measurement [50–53]. Since neither DCT-IOP, IOPcc, nor bIOP showed a significant correlation to CCT, all measurement devices can be used for correction of a possible CCT influence.

On the other hand, results concerning age dependency are inconsistent. Joda et al. reported a weak age dependency of uncorrected CST-IOP in a study with a high case number. According to this investigation, an algorithm correcting CST-IOP for CCT and age is mandatory [30]. The implementation of bIOP resulted in a significant reduction of the correlation of IOP and participants' age and CCT [30]. In contrast to these findings, in the present study, we observed a weak but significant age dependency of bIOP. This is in accordance with results published by Vinciguerra et al., who reported bIOP discrepancies in different age groups with slightly lower bIOP with increasing age [29]. In this sense, a negative age correlation was detectable in our study. This association needs consideration. With higher age, cornea tends to be stiffer which might be caused by age-induced reduction of matrix components [29,53–57] and potentially leads to IOP overestimation by GAT in older persons. However, corneal stiffness might not be reasonable for the correlation between age and bIOP because a positive association would be expected. Furthermore, with increasing age, corneal thickness falls [58] and a reduced CCT is associated with false high IOP measurement [26]. Indeed, bIOP calculation formula contains correction for CCT. Therefore, thickness increase also may not be the reason for the positive association between age and bIOP. A further reason could be corneal topography or thickness distribution. An increased keratometry with aging takes the cornea to a more prolate shape [58] which also might influence bIOP measurement.

Looking at age dependency of uncorrected CST-IOP, results are inconsistent. Some authors found no age correlation of CST-IOP [1, 59] while others reported a weak age influence [30]. Nevertheless, in the studies using uncorrected Corvis-IOP by Matsuura et al., only patients with preexisting primary open-angle glaucoma, a higher mean age (63.7 ± 10.1 years), and a smaller age range (41–86 years) were included [1]. Also, Nemeth and colleagues included participants with a higher mean age (61.24 ± 15.72 years) [59]. According to the results of earlier studies, corneal biomechanical parameters measured by CST seem to be age-dependent [29]. Furthermore, glaucoma [60] and antiglaucomatous medication [61] might induce changes in corneal biomechanics. These differences of the included subjects might explain the deviating results between earlier findings of CST-IOP [1, 59] and bIOP results by Vinciguerra et al. [45] and our study. However, even though the small mean difference between bIOP and GAT-IOPc might lead to the assumption to favour bIOP as a noncontact parameter in daily practice, the observed age correlation questions the superiority of this parameter.

An important criterion for the quality of IOP measurement represents the stability of results with repeated measurements. Tejwani et al. reported a lower coefficient of variation for CST-IOP (4.61%) compared to DCT (7.18%) and IOPcc (11.06%) [17]. In addition, recent studies by Nemeth et al. and Reznicek et al. showed a high repeatability of IOP values measured with CST [49, 59]. In the present study, no repeated IOP measurements were performed. Furthermore, beside CCT and age, corneal biomechanical properties might exert an influence on IOP readings. However, material properties were not investigated separately in this study. A further limitation might be IOP falsification by consecutive application of different tonometers. However, Tejwani et al. did not find an influence of sequential measurements using GAT, DCT, ORA, and CST with a break between the single measurements of only 5 minutes [17]. Hence, it can be assumed that a break of 10 minutes, as it was used in our study, might be enough time to rule out falsifications.

In conclusion, all used tonometry devices revealed a good consistency of IOP results with GAT-IOPc. Except GAT-IOPc, no influence of CCT was detectable so that the used correction algorithms appear to be appropriate to compensate varying corneal thickness. The highest accordance was found between GAT-IOPc and bIOP. Nevertheless, as only this parameter showed an age correlation, the superiority of the bIOP remains questionable. Further studies are certainly needed to elucidate the role of bIOP for IOP measurement.

## Figures and Tables

**Table 1:** Descriptive data of participants and results of intraocular pressure (IOP) measurements (mean value ± standard deviation).

Descriptive data	
Number of subjects	94
Eye (right/left)	47 (50%)/47 (50%)
Gender (female/male)	39 (41.5%)/55 (58.5%)
Mean age (years)	45.6 ± 17.2
CCT (*μ*m)	553 ± 33
Intraocular pressure	
GAT-IOPc (mmHg)	12.9 ± 2.4
DCT-IOP (mmHg)	16.1 ± 2.6
IOPcc (mmHg)	15.6 ± 3.3
bIOP (mmHg)	13.5 ± 2.4

GAT-IOPc: corrected Goldmann applanation tonometry-IOP; DCT-IOP: dynamic contour tonometry-IOP; IOPcc: corneal compensated IOP by using ocular response analyzer; bIOP: biomechanical corrected IOP by using Corvis ST; CCT: central corneal thickness.

**Table 2:** Amount of mean difference (±standard deviation, in mmHg) of the results of intraocular pressure (IOP) measurement using different tonometers.

	DCT-IOP	IOPcc	bIOP
GAT-IOPc	3.2 ± 2.3, *P* < 0.001	2.7 ± 3, *P* < 0.001	0.6 ± 2.7, *P*=0.146
DCT-IOP	—	0.5 ± 2.4, *P*=0.377	2.6 ± 2.5, *P* < 0.001
IOPcc	—	—	2.1 ± 2.9, *P* < 0.001

GAT-IOPc: corrected Goldmann applanation tonometry-IOP, DCT-IOP: dynamic contour tonometry-IOP, IOPcc: corneal compensated IOP by using ocular response analyzer, bIOP: biomechanical corrected IOP by using Corvis ST.

## Data Availability

The data used to support the findings of this study are available from the corresponding author upon request.
